# Comment on Lecca, L.I.; Portoghese, I.; Mucci, N.; Galletta, M.; Meloni, F.; Pilia, I.; Marcias, G.; Fabbri, D.; Fostinelli, J.; Lucchini, R.G.; Cocco, P.; Campagna, M. Association between Work-Related Stress and QT Prolongation in Male Workers. *Int. J. Environ. Res. Public Health* 2019, *16*, 4781

**DOI:** 10.3390/ijerph17020461

**Published:** 2020-01-10

**Authors:** Javad Alizargar, Nan-Chen Hsieh, Shu-Fang Vivienne Wu, Shih-Yen Weng

**Affiliations:** 1 Research Center for Healthcare Industry Innovation, National Taipei University of Nursing and Health Sciences, Taipei City 112, Taiwan; 2 Department of Information Management, National Taipei University of Nursing and Health Sciences, Taipei City 112, Taiwan; nchsieh@ntunhs.edu.tw; 3 College of Nursing, School of Nursing, National Taipei University of Nursing and Health Sciences, Taipei City 112, Taiwan; shufang@ntunhs.edu.tw

## Abstract

Lecca et al., in a recent publication in the *Int. J. Environ. Res. Public Health* had made some mistakes in the statistical analysis and interpretation of the results. Age was not a clear contributing factor in the prolongation of QT interval in the electrocardiogram (ECG), as there were strong confounders in their study. The effects of age were mainly faded out because of the age range of the participants. The use of Pearson’s correlation is questionable because of the normality assumptions was not met on the studied variables. They also made some conclusions about the effects of long and night shifts on the QT prolongation that were not appropriate based on their study type. All of these mentioned issues might completely change the validity of the conclusions they made.

We read with interest the article by Lecca et al. [1], in which the authors focused on the effects of work-related stress on changes in the QT interval in the routine electrocardiogram (ECG) among workers without overt cardiovascular dysfunction. They also tried to consider some confounders in their analysis. However, certain concerns have not been addressed properly, which could change some conclusions they made.

As previously described [2], the QT increases with some risk factors, including electrolyte disturbances, female gender, cardiovascular diseases, genetic variants, and using certain drugs. However, the main factor is age. Lecca et al. [1] had excluded the people that had some of the these conditions so they could not interfere with the conclusion of their study. They also showed that the observed effects of age in the correlation analysis disappeared when they controlled for some factors, such as operating location, physical activity, systolic blood pressure (SBP), age, education, and alcohol intake; yet, they concluded that “age was a clear contributing factor in QTc and QTi prolongation in our study”, and compared their results to other studies. This statement is wrong, as the effects of age are the result of the operating location and physical activity, but as there are borderline *p*-values of 0.048 and 0.047 in Sites 1 and 2, respectively, between physical activity and QTc, we can assume that the main confounder can be considered to be the site location. Unfortunately, they did not include all of the risk factors like duration of employment in the multivariate analysis; hence, the conclusion about the main risk factor affecting QTc is questionable. There is a significant (*p* = 7.6 × 10^−7^) difference in age between the two sites, so the conclusion about the effects of age and site location on QTc requires more detailed information and analysis. Providing information about the age range of the study participants might be useful for explaining why age has not shown an independent effect on QTc. Workers before retirement can be considered as young to middle age, so increased age can be ruled out as a main risk factor.

The other main concern about this study is that they performed a Pearson’s correlation analysis and built the majority of their conclusions based on this before analyzing the assumptions that should have been made before such an analysis. Correlation has been widely used to quantify the association between two paired numeric variables. Correlation can show how strong a linear relationship between two variables is. If both variables are normally and if the normality assumption is not met, distributed a Pearson’s correlation and a Spearman’s correlation analysis, respectively, are appropriate [3]. Lecca et al. [1] provided no information about the normality of the variables, and it is very possible that some of their variables were not normally distributed, yet they performed a Pearson’s correlation on them.

The next issue is that in their study, the Site 2 workers had longer QTc intervals than or Site 1, and they concluded that this was because of the fact that the Site 1 workers were more frequently engaged in long shifts and night shifts. This conclusion has some problems. First, the two groups are not similar in some key aspects, such as age and the duration of employment. This is also not a matched case control study for such a conclusion. Second, workers with subclinical cardiovascular disease might choose not to take long and night shifts. So, the fact that the Site 2 workers had a prolonged QTc interval might be because they are healthier than the Site 1 workers, and not because the long and night shift working caused a prolong QTc interval.
